# Intravenous administration of LPS activates the kynurenine pathway in healthy male human subjects: a prospective placebo-controlled cross-over trial

**DOI:** 10.1186/s12974-021-02196-x

**Published:** 2021-07-17

**Authors:** Vincent Millischer, Matthias Heinzl, Anthi Faka, Michael Resl, Ada Trepci, Carmen Klammer, Margot Egger, Benjamin Dieplinger, Martin Clodi, Lilly Schwieler

**Affiliations:** 1 Department of Molecular Medicine and Surgery (MMK), Karolinska Institutet, Stockholm, Sweden; 2 Translational Psychiatry, Center for Molecular Medicine, Karolinska University Hospital, Stockholm, Sweden; 3 Department of Psychiatry and Psychotherapy, Medical University of Vienna, Vienna, Austria; 4 Department of Internal Medicine, Konventhospital Barmherzige Brueder (St. John of God Hospital), Seilerstaette 2, 4021 Linz, Austria; 5 ICMR–Institute for Cardiovascular and Metabolic Research, JKU Linz, Linz, Austria; 6 Department of Physiology & Pharmacology, Sec. Neuropsychoimmunology, Karolinska Institutet, Stockholm, Sweden; 7 Department of Laboratory Medicine, Konventhospital Barmherzige Brueder (St. John of God Hospital), Linz, Austria

**Keywords:** Lipopolysaccharides (LPS), Experimental endotoxemia, Kynurenine metabolites, Inflammation

## Abstract

**Background:**

Administration of lipopolysaccharide (LPS) from Gram-negative bacteria, also known as the human endotoxemia model, is a standardized and safe model of human inflammation. Experimental studies have revealed that peripheral administration of LPS leads to induction of the kynurenine pathway followed by depressive-like behavior and cognitive dysfunction in animals. The aim of the present study is to investigate how acute intravenous LPS administration affects the kynurenine pathway in healthy male human subjects.

**Methods:**

The present study is a prospective, single-blinded, randomized, placebo-controlled cross-over study to investigate the effects of intravenously administered LPS (Escherichia coli O113, 2 ng/kg) on tryptophan and kynurenine metabolites over 48 h and their association with interleukin-6 (IL-6) and C-reactive protein (CRP). The study included 10 healthy, non-smoking men (18–40 years) free from medication. Statistical differences in tryptophan and kynurenine metabolites as well as associations with IL-6 and CRP in LPS and placebo treated subjects were assessed with linear mixed-effects models.

**Results:**

Systemic injection of LPS was associated with significantly lower concentrations of plasma tryptophan and kynurenine after 4 h, as well as higher concentrations of quinolinic acid (QUIN) after 48 h compared to the placebo injection. No differences were found in kynurenic acid (KYNA) or picolinic acid plasma concentrations between LPS or placebo treatment. The KYNA/kynurenine ratio peaked at 6 h post LPS injection while QUIN/kynurenine maintained significantly higher from 3 h post LPS injection until 24 h. The kynurenine/tryptophan ratio was higher at 24 h and 48 h post LPS treatment. Finally, we report an association between the kynurenine/tryptophan ratio and CRP.

**Conclusions:**

Our findings strongly support the concept that an inflammatory challenge with LPS induces the kynurenine pathway in humans, activating both the neurotoxic (QUIN) and neuroprotective (KYNA) branch of the kynurenine pathway.

**Trial registration:**

This study is based on a study registered at ClinicalTrials.gov, NCT03392701. Registered 21 December 2017.

**Supplementary Information:**

The online version contains supplementary material available at 10.1186/s12974-021-02196-x.

## Introduction

Lipopolysaccharide (LPS) is a component from the cell wall of Gram-negative bacteria that binds to toll-like receptor 4 and triggers secretion of pro-inflammatory cytokines in a dose-dependent manner [1, 2]. LPS is broadly used in animal and cell studies and reflects parts of the complex host-pathogen interaction of a bacterial infection. LPS-induced inflammation, or artificial endotoxemia, is an established model for experimental, systemic inflammation in humans. It reliably causes a febrile, systemic inflammatory response in healthy individuals and is the most widely accepted model to study the pathophysiology of human host response to infection [3]. Studies in humans show that endotoxemia induces sickness behavior [4–8], depressed mood [9–12], alters pain perception [13], reduces motivation [14], and affects the communication between the immune system and the brain [15].

**Fig. 1:**
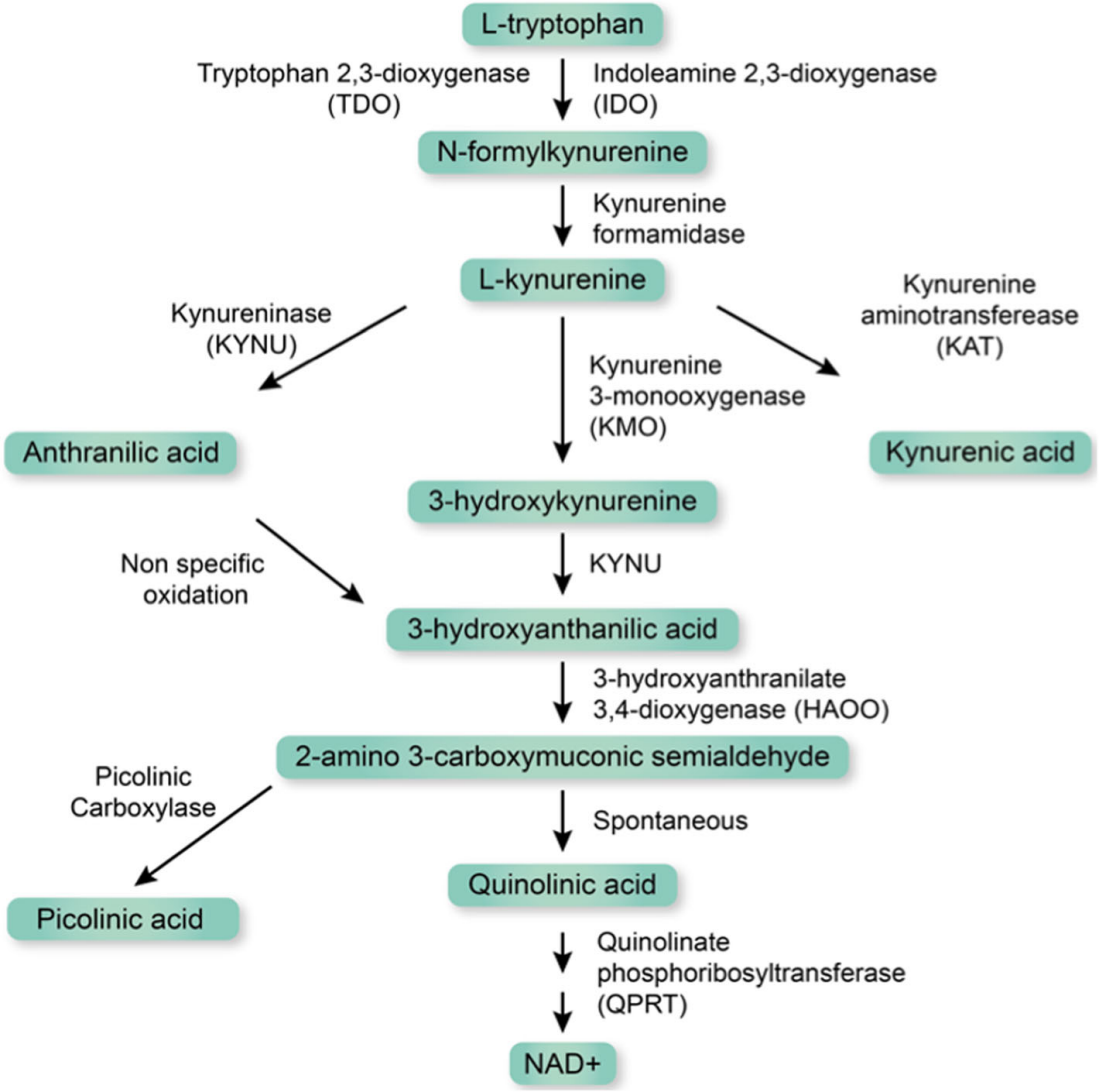
Schematic overview of the kynurenine pathway. The first and rate-limiting step is catalyzed by tryptophan 2,3-dioxygenase (TDO2) or by indoleamine 2,3-dioxygenase (IDO)1 and 2. N-formyl kynurenine is then converted by kynurenine formamidase to l-kynurenine before entering different possible branches, depending on cell-type or environmental context, to form various metabolites, which can exhibit immunological, antioxidant, or neurological activities. Pathways represented by two arrows involve several metabolites and enzymatic reactions. Nicotinamide adenine dinucleotide (NAD+)

It is well known that the activation of the immune system initiates the kynurenine pathway (Fig. 1) and thereby induces tryptophan degradation [16]. The two rate limiting enzymes of this pathway are tryptophan 2,3-dioxygenase (TDO2) [17] and indoleamine-pyrrole 2,3-dioxygenase (IDO1) [18]. TDO2 is mainly regulated by corticosteroids and glucagon [19], but can also be induced by immune activation [20] and more specifically by interleukin (IL)-1β [21, 22]. IDO1 is expressed in different immune cells such as monocytes, dendritic cells, and macrophages, and its activity and expression is strongly associated with inflammatory stimuli [23]. IDO1 gene and protein expression is induced by a number of inflammatory cytokines, such as interferon (IFN)-γ, tumor necrosis factor (TNF)-*α* [24, 25], TNF-β [26], IL-1β [27], IL-2 [28], IL-6 [29], IL-27 [30], and IL-10 [31]. While the regulation of IDO1 through the janus kinase 1 and 2 and signal transducer and activator of transcription 1 system is well established [32, 33], less is known about the regulation of TDO2 by immune activation. Recent studies in glioblastoma cells, however, suggest that IL-1β activates TDO2 via the CCAAT-enhancer-binding protein (C/EBP)-β in a mitogen-activated protein kinase dependent fashion [34].

Activation of the kynurenine pathway in connection with bacterial infection has been suggested to fine-tune the suppression of microorganisms via the depletion of intracellular tryptophan [35] and local tryptophan depletion in host cells blunts immune reactions via an anti-proliferative and a pro-apoptotic effect on T cells [36]. However, kynurenine metabolites do not only affect the immune system, but are also seen as neuromodulators. Indeed, the stimulation of the kynurenine pathway results in the production of several neuroactive metabolites such as kynurenic acid (KYNA) and quinolinic acid (QUIN). KYNA, mainly produced by astrocytes, acts as an N-Methyl-D-aspartate (NMDA) and alpha7 nicotinic acetylcholine receptor (α7nAChR) antagonist and has neuroprotective properties [37–39]. QUIN, on the other hand, is mainly synthesized by microglia and macrophages [40]. It acts as an agonist at NMDA receptors [39] and has been shown to have excitotoxic properties [41]. The kynurenine pathway has therefore been suggested as a potential link between the immune system and brain physiology [42]. Indeed, the link between the immune system, the kynurenine pathway and aberrant behavior is well established in animal models. For example, treatment with LPS induces IDO1 expression [43, 44] along with sickness behavior [42], depression-like behavior [44], and cognitive deficits [45–47]. Finally, clinical studies clearly support a link between the immune system, the kynurenine pathway and neuropsychiatric symptoms such as depression [48], psychotic symptoms [21, 48], and cognitive deficits [49].

The aim of the present study is to investigate whether acute, intravenous LPS administration, given in a dose evoking a substantial inflammatory response [50] affects the plasma concentration of kynurenine metabolites in healthy human subjects.

## Materials and methods

### Subjects

Twenty-four volunteers were screened between January and July 2018 by physical examination, routine laboratory testing, and an electrocardiogram. Of those, 8 withdrew and 6 were excluded (2 because of Factor V Leiden, 1 because of increased homocysteine, 2 because of increased Lupus antibodies, 1 because of microcythemia). The 10 included individuals were non-smoking, free from medication or substance abuse, without any known history of relevant disease. Their mean age was 24.1 (standard deviation, 3.7) years, and the average body mass index was 25.2 (standard deviation, 1.6) kg/m^2^. ECG of all participants showed sinus rhythm with normal repolarization. Given the cross-over design, all 10 individuals acted as their own controls, receiving both the placebo and LPS injection on 2 different occasions, the order being randomly assigned.

### Study protocol

The present study is a prospective, single-blinded, randomized, placebo-controlled cross-over study. Participants were advised not to drink coffee 24 h before each study day and to fast overnight. During the study day, they were allowed to drink 1.5 L of non-sparkling mineral water and eat after completion of the 6 h blood sampling.

Intravenous catheters (B. Braun) were inserted into a vein on each arm (separate catheters for infusion and blood sampling). Subjects were given 0.9% saline (placebo) and LPS (*Escherichia coli* O113) at a dose of 2 ng/kg in a cross-over design on two different study days, separated by a washout period of at least 14 days. LPS or placebo treatment were given intravenously over 5 min together with 0.9% saline over 90 min [200 ml/h]) on the respective day. EDTA-blood samples were taken before infusion of LPS or placebo and following infusion, at 15, 30, 45, 60, 90, 120, 180, 240, and 360 min as well as 24 h and 48 h after infusion. The last two samples of blood were taken at 8:00 a.m. after overnight fasting. During the study day, subjects rested in a supine position and were monitored continuously (electrocardiogram, heart rate, non-invasive blood pressure, and temperature) (Supplementary Table 1).

Blood samples were centrifuged immediately after blood sampling and plasma samples were stored at – 80 °C. For the quantification of kynurenine metabolites, plasma aliquots were sent on dry ice to Karolinska Institutet, where they were stored at − 80 °C until further analysis.

### Preparation of LPS

The purified LPS was prepared from *Escherichia coli* O113 (National Reference Bacterial Endotoxin; lot #94332B1, Investigational Drug Management at the National Institutes of Health (NIH), Bethesda, MD) and stored according to good clinical practice guidelines. Endotoxin was supplied in vials as a sterile, white, lyophilized powder, with each vial containing 10,000 endotoxin units (1 μg). Before infusion, endotoxin was reconstituted with sterile water and prepared according to the recommendations of the manufacturer.

### Analysis of IL-6 and C-reactive protein (CRP)

IL-6 was determined with a chemiluminescent microparticle immunoassay on a Cobas e411 (Roche Diagnostics). CRP was directly measured with standard assays on an Architect c16000 analyzer (Abbott Diagnostics). Both IL-6 and CRP data have been reported earlier [50].

### Analysis of kynurenine metabolites using UPLC-MS/MS

Tryptophan, kynurenine, KYNA, 3-hydroxykynurenine (3-HK), QUIN, and picolinic acid were analyzed with a Xevo TQ-XS triple quadrupole mass spectrometer (Waters, Manchester, UK) equipped with a Z-spray electrospray interface and a Waters Acquity UPLC I-Class FTN system (Waters, Milford, MA) using a validated method previously described [51].

#### Sample preparation

Thirty microliters of human EDTA-plasma, calibrator, or quality control sample were mixed with 30 μl of internal standard solution (IS; 0.5 μM in 10% ammonium hydroxide, UPLC grade) solution for 15 seconds. Then 60 μl of 200 nM ZnSO_4_ (5 °C) was added and mixed for 15 s before 30 μl of methanol (5 °C) (UPLC grade) were added and mixed for 15 s. The mixture was then centrifuged for 10 min at 2841×*g* at room temperature. Thirty microliters of the supernatant were mixed with 30 μl of formic acid 5% in LC-MS Certified Clear Glass 12 × 32 mm vials (Waters, product no. 186005662CV) before transfer to an autosampler (set to 5 °C), which injected 1.5 μl per sample into the UPLC–MS/MS system.

#### Chemicals

Normal standards—tryptophan, l-kynurenine, pyridine-2,3-dicarboxylic acid (QUIN), KYNA, picolinic acid, 3-HK—were purchased from Sigma-Aldrich (MO, USA). The Internal standards (IS)—tryptophan-d_3_, l-kynurenine-d_4_, QUIN-d_3_ [13C6], 3-HK-d_3_—were purchased from Toronto Research Chemicals Canada (Toronto Canada). KYNA-d_5_ and picolinic acid-d_4_ were obtained from C/D/N Isotopes Inc. (Quebec, Canada). Solutions for the mobile phases—water, methanol and formic acid 99%—were all LC-MS grade from Chromasolve, Honeywell, VWR International AB, Stockholm (Sweden). Solutions for plasma preparations: ammonia (32%) was purchased from VWR and ZnSO_4_ was purchased from Sigma-Aldrich (MO, USA). Stock solutions of all unlabeled standards (tryptophan, kynurenine, QUIN, KYNA, picolinic acid, and 3-HK) were prepared in water for HPLC, LCMS grade, and stored at – 20 °C. Calibrators were generated mixing all compounds in a final solution of 8.3 μM and 10 times higher for tryptophan, 83 μM.

#### Analysis with UPLC–MS/MS

The UPLC–MS/MS system was operated in electrospray positive multiple reaction monitoring (MRM) mode. The conditions were set as follows for the interface: source temperature of 150 °C; desolvation gas flow rate 1000 L/h; cone gas flow rate 150 L/h; capillary voltage of 3.0 kV; desolvation temperature 650 °C; detector gain 1. The UPLC condition was as follows: column, Acquity HSS T3 1.8 μm with dimensions 2.1 × 150 mm, (Waters, part number: 186003540) column temperature 50 °C; guard column (Waters, Vanguard HSS T3 1.8 μm 2.1 × 50 mm column, part number: 186003976) was installed to retain contaminants from the mobile phase. The mobile phase A was 0.6% formic acid in water (UPLC grade) and the mobile phase B was 0.6% formic acid in methanol (UPLC grade). The flow rate was 0.3 ml/min and the run time for each sample was 13.0 min. The autosampler was set at 4 °C. Data processing was performed using Masslynx 4.1 software. The software was used for calculating the dwell times for the MRM channels, giving a desired number of 15–20 data points across the chromatographic peak.

The m/z for the MRM transitions of each individual analyte, along with optimal cone voltages and collision energies were determined by manual tuning using the instrument’s built-in fluidics system (Masslynx 4.1 software). The MRM transition providing the highest sensitivity was chosen as quantification trace for all compounds, except for tryptophan and kynurenine where the C13 isotopes were selected to reduce overall signal intensity. The intensities of peaks in selected MRM transitions were recorded at previously determined retention times (rt) and optimized instrumental settings were as follows: tryptophan m/z 206→118 and 146 at rt 7.01 min, tryptophan-d_3_ m/z 208.1→118.8 at rt 7.0 min; kynurenine m/z 209→94 and 146 at rt 5.76 min, kynurenine-d_4_ m/z 213→94 at rt 5.7 min; KYNA m/z 190.1→116 and 144 at rt 7.99 min, KYNA-d_5_ m/z 195→121 at rt 7.89 min; 3-HK m/z 225.2→110.1 and 162.1 at rt 4.1 min, 3-HK-d_3_ m/z 228.2→163 at rt 3.78 min; QUIN m/z 168.1→78 and 124 at rt 2.94 min; QUIN-d_3_ m/z 171→81 at rt 2.86 min; picolinic acid m/z 123.9→78 and 96 at rt 2.17 min; picolinic acid-d_4_ m/z 128→82 at rt 2.14 min).

The 240 samples (24 per subject) were divided into three batches. All samples from the same subject (placebo and LPS treated) were assayed in the same batch and 5% of all samples in each batch were quality controls (QC, spiked plasma), serving to monitor for significant differences between batches and between duplicates. Each batch included ten standards (five dilutions, each in duplicate ranging from 0.006 to 8.3 μM, tryptophan 0.06–83 μM), prepared exactly as plasma, with the same number of internal standards. The data from the standards was used to construct standard curves and the analyte content of each sample was interpolated from the respective standard curves. All quality controls and standards were run in duplicate, and the intra-assay coefficients of variation (CV) within all batches were less than 7%. The accepted inter-assay CV was 15%. All samples were higher than the limit of quantification (LOQ signal-to-noise ratio of ten). LOQ was 0.006 μM for tryptophan, kynurenine, KYNA, and QUIN and 0.01 μM for 3-HK and picolinic acid. We could successfully detect 98% of all samples. Five KYNA values, six QUIN values, one picolinic acid value, six tryptophan values, and three 3-HK values were removed due to a %CV that was higher than 15% between QC duplicates.

### Statistical analyses

**Fig. 2:**
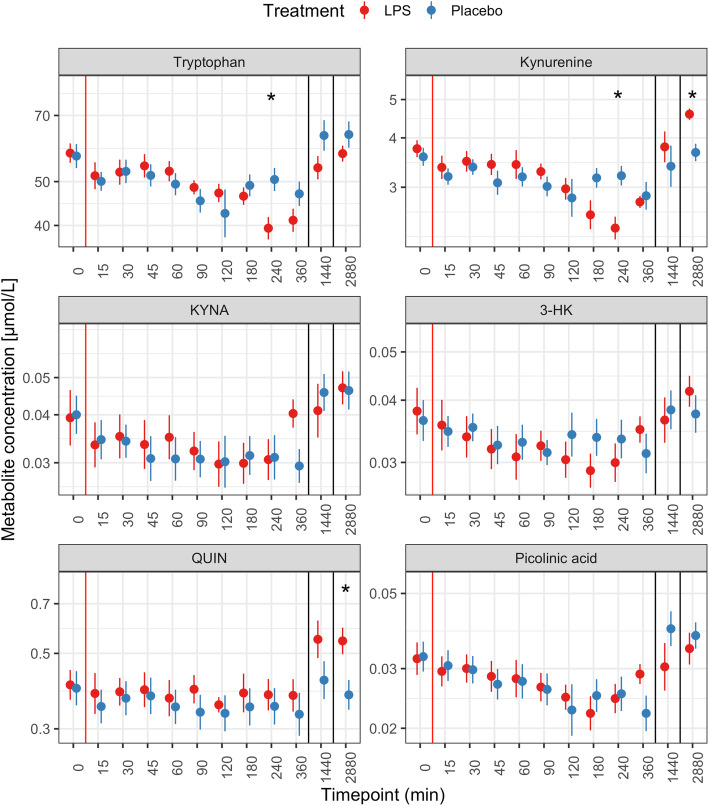
Kynurenine metabolite levels after LPS (red) or placebo (blue) injection. The red line indicates the injection, the black lines the change between the three consecutive days. Data is presented as mean and standard error of the mean. *p < 0.005 (statistically significant results). KYNA: kynurenic acid, 3-HK: 3-hydroxykynurenine, QUIN: quinolinic acid

**Fig. 3:**
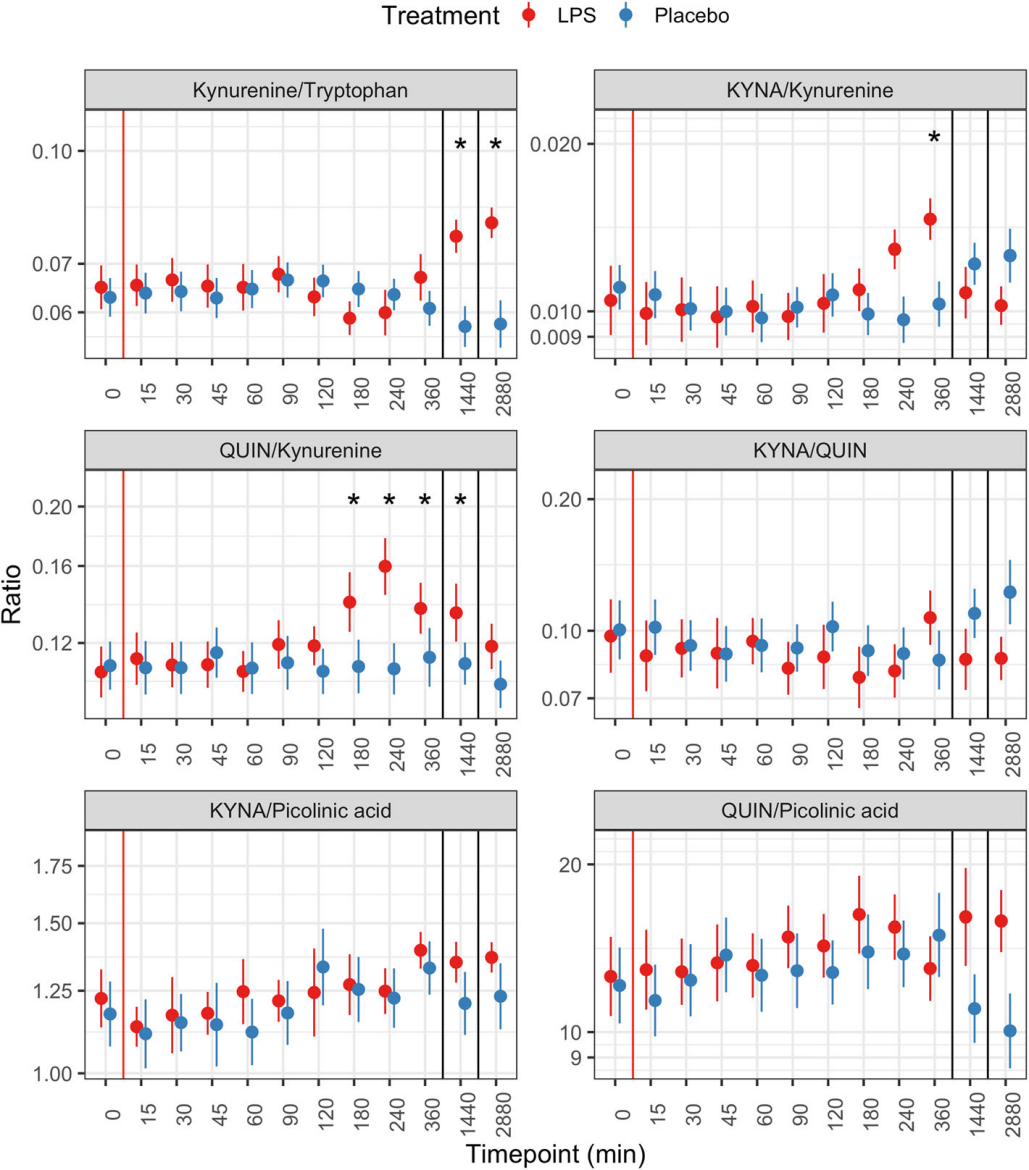
Ratios between metabolites after LPS (red) or placebo (blue) injection. The red line indicates the injection, the black lines the change between the three consecutive days. Data is presented as mean and standard error of the mean. *p < 0.005. KYNA: kynureninic acid, QUIN: quinolinic acid

Because of the skewed distributions at several timepoints, we performed a log-transformation of all metabolite levels in order to normalize the distribution and reduce the number of influential points. Ratios were calculated on the non-transformed values and then log-transformed in order to consider proportional rather than absolute changes in the dependent variable (i.e., doubling or halving of the ratio are equidistant following the log-transformation). The y-axes of the figures presenting this data (Figs. 2 and 3) are therefore in the log-scale.

Differences in kynurenine metabolites and ratios between the LPS treatment and placebo were assessed as follows: for each metabolite or ratio and each timepoint, a linear mixed-effects model was performed with log(metabolite levels) or log(ratios) entered as dependent variable, treatment condition, and baseline metabolite levels or baseline ratio levels, respectively, entered as fixed-effects, and participant-id as random-effect. The alpha level of significance was set at 0.05. There was a significant pairwise correlation within the twelve tested metabolites and ratios, violating the assumption of test independence. We therefore estimated the number of effective tests neff as described by Cheverud and Nyholt [52, 53] based on the overall correlation between metabolites and ratios using the R packages poolr [54]. We adjusted *p* values to account for the 10 effective independent tests using Bonferroni correction; an alpha level of 0.005 was therefore considered statistically significant.

To assess associations between the ratios that showed an increase upon LPS stimulation (kynurenine/tryptophan, KYNA/kynurenine, QUIN/kynurenine) and the inflammatory markers (IL-6 and CRP), linear mixed effects models were performed, with log(ratios) entered as dependent variable, log(IL-6) or log(CRP) as fixed-effects, and participant-id as random-effect. CRP values equal to 0 were replaced with 0.01 (1/10 of the CRP detection limit) to allow for log-transformation. The alpha-level of significance was set at 0.05. Bonferroni correction for 6 tests was applied and p values < 0.0083 were considered significant.

To assess the association between the increase in inflammatory markers and the increase in ratios after LPS treatment, the difference between the highest level and baseline (deltamax) were calculated for CRP, IL-6, and the metabolite ratios. Correlations between deltamax(IL6) or deltamax(CRP) and deltamax(ratios) were assessed using Pearson’s correlation coefficient.

Statistical analyses were conducted using R programming language (R version 4.0.2) [55], including the packages nlme [56]. Graphs were created using the package ggplot [57].

## Results

Systemic injection of LPS was associated with significantly lower concentrations of tryptophan and kynurenine at the 240 min (4 h) timepoint compared to placebo (Fig. 2, top left and top right panel). Forty-eight hours after administration, kynurenine and QUIN were higher in the LPS treated condition compared to the placebo-treated condition (Fig. 2, top right and bottom left panel). Concentrations of KYNA, picolinic acid, and 3-HK were not significantly different between the two groups at any time-point (Fig. 2).

The ratios kynurenine/tryptophan, KYNA/kynurenine, and QUIN/kynurenine showed significant increases after LPS injection. The kynurenine/tryptophan ratio was higher at 24 h and 48 h post injection. The KYNA/kynurenine ratio peaked at 6 h post injection, while QUIN/kynurenine was significantly higher from 180 min post injection until 24 h (Fig. 3).

**Fig. 4:**
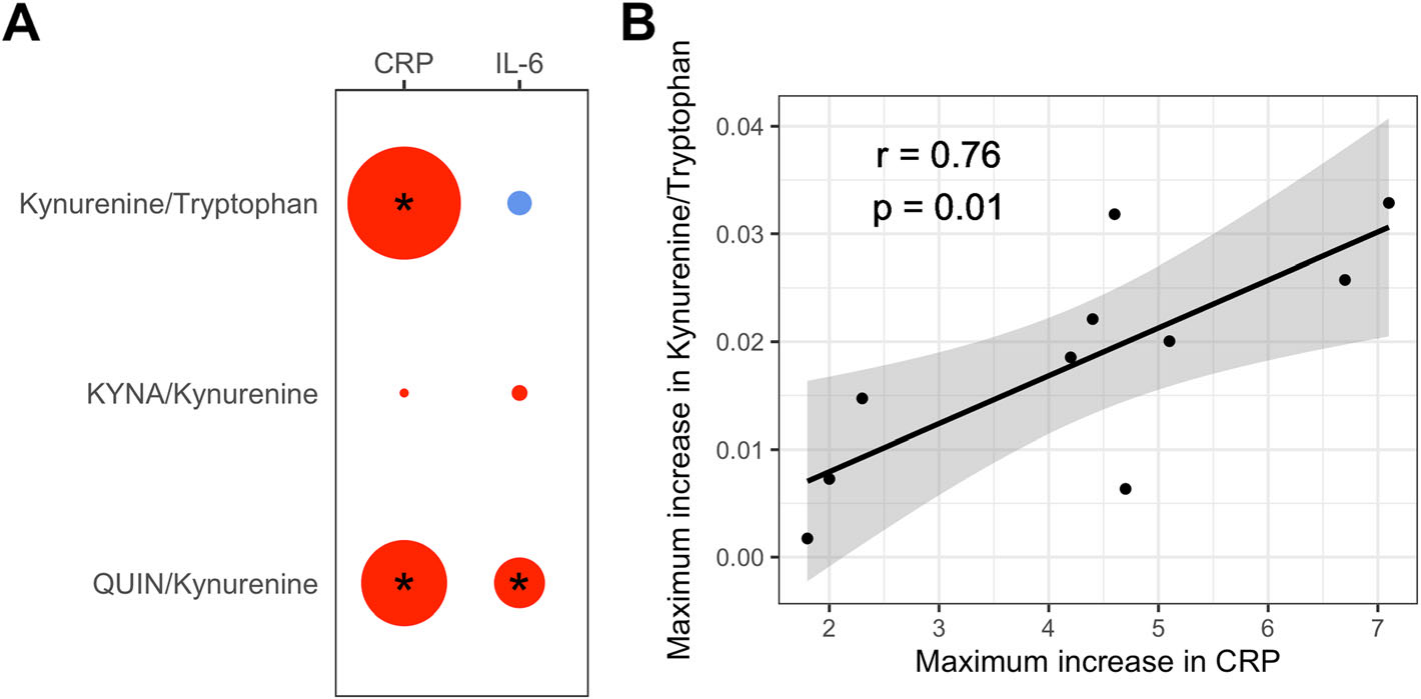
Associations between **A** metabolite ratios and CRP or IL-6 over all timepoints. The size of the circles indicates the strength of the association (−log_10_p), the color indicates the direction of the association (red: positive, blue: negative). *p < 0.0083. KYNA: kynureninic acid, QUIN: quinolinic acid. **B** Correlation between the maximum increase in CRP (mg/dL) and the maximum increase in the kynurenine/tryptophan ratio independent of time. The black line indicates the conditional mean, and the grey area indicates the 95% confidence interval

Among the ratios that showed significant increases after LPS injection, the ratio kynurenine/tryptophan showed a positive association with CRP (*p* = 6.4 × 10^−16^), while the ratio QUIN/kynurenine showed a positive association with CRP (*p* = 5.1 × 10^−9^) and IL-6 (*p* = 2.9 × 10^−3^) over all timepoints (Fig. 4A, Supplementary Figure 1). Analyzing each timepoint separately, CRP showed the strongest association with the ratio kynurenine/tryptophan at 1440 min when plasma levels of CRP peaked (p = 0.0009), while the IL-6 was only associated at nominal significance with the ratio QUIN/Kynurenine at its peak at 180 min (p = 0.02) (Supplementary Figure 2). When specifically assessing the association between the maximum increase in inflammatory markers and the maximum increase in ratios after LPS stimulation, only the maximum increase in ratio kynurenine/tryptophan and the maximum increase in CRP showed a nominally significant correlation (r = 0.76, p = 0.01) (Fig. 4B). Although the correlation between the maximum increases of CRP and IL-6 after LPS stimulation was highly significant (r = 0.81, *p* = 0.004), the correlation between the maximum increase in kynurenine/tryptophan and the maximum increase in IL-6 was not significant (r = 0.61, *p* = 0.06).

## Discussion

In the present study, the kinetics of kynurenine metabolites following an intravenous administration of LPS in 10 healthy subjects were investigated. Experimental endotoxemia was found to activate both the neurotoxic and the neuroprotective branch of the kynurenine pathway. Our results point to an activation of the whole pathway after LPS treatment, as indicated by enhanced ratios of kynurenine/tryptophan, QUIN/kynurenine, and KYNA/kynurenine, as approximations for the enzymatic activity of IDO1/TDO2, KMO/Kynureninase (KYNU), and KATs, respectively. Our data suggest that LPS injection increases the activity of the downstream enzymes (KMO/KYNU and KAT) first, peaking within 3–6 h, leading to a depletion of both tryptophan and kynurenine 4 h post-administration. The activity of the upstream rate-limiting enzymes (IDO1/TDO2) increased only after 24–48 h.

We have previously reported that systemic injection of LPS induces an inflammatory response in these subjects with a rapid increase of IL-6 (highest levels detected at 3 h), and a slower increase of CRP reaching maximal detection after 24 h after LPS injection [50]. Here, we found associations between the metabolite ratios and levels of these inflammatory markers, indicating an induction of the kynurenine pathway driven by LPS induced immune activation. Interestingly, CRP has been found to be a marker of peripheral immune activity involving both TNF and IL-1*β* [58], cytokines inducing the two rate limiting enzymes of the kynurenine pathway.

Two studies on the effects of experimental endotoxemia on the kynurenine pathway have previously been performed in humans [12, 59]. Our study complements these results by being the first using a placebo-controlled, cross-over design over a period of 48 h with high temporal resolution over the first 6 h. Padberg and colleagues showed that injection of LPS at 4 ng/kg induces the turnover of kynurenine by activation of IDO1, as measured by the ratio kynurenine/tryptophan, 6–8 h post LPS injection [59]. In the present study, using only half of the LPS dose, we show that the turnover of kynurenine is enhanced up to 48 h post LPS injection. In a larger study, 115 adults were randomized to receive either LPS (0.8 ng/kg body weight, *Escherichia coli* group O:113) or placebo [12]. LPS treatment led to an induction of the kynurenine pathway, as measured by the increased kynurenine/tryptophan ratio, a decrease in plasma tryptophan, and an increase in plasma kynurenine and KYNA at 2 h and 6 h, respectively, post injection. Even though we did not observe any rapid increase in the concentration of kynurenine or its metabolites in the present study, we confirm a decrease in tryptophan concentration at 4 h. Furthermore, we also observed an induction of the pathway, reflected by the altered kynurenine/tryptophan ratio, even if it appeared slightly later, after 24 and 48 h.

With an increasing body of evidence linking depression and inflammation, the kynurenine pathway has been brought forward as one possible link between them. Increased kynurenine metabolites and kynurenine/tryptophan ratio have been associated with both the onset of depression and depression severity in medically ill patients receiving the inflammatory cytokine IFN-α [60]. Interestingly, pre-clinical studies show that IDO activity after systemic immune stimulation is necessary for the manifestation of depression-like behavior [61]. Furthermore, several metabolites in the kynurenine pathway such as anthranilic acid [62], KYNA, and QUIN [63] have been suggested as potential biomarkers for depression. However, even if a growing body of literature points to an involvement of the neurotoxic kynurenine metabolite QUIN [48], the precise role of kynurenine metabolites in the causation of treatment-induced depression is still unclear [64–66].

From this psychiatric point of view, the lack of quantitative behavioral data is a clear limitation. Even if all subjects except one experienced flu-like symptoms after LPS infusion, peaking between 60 and 90 min and resolving after 300 min, these symptoms were not quantified in all individuals. We therefore could not investigate whether changes in plasma concentrations of tryptophan or kynurenine metabolites correlate with sickness behavior or depression mood, as suggested by other studies. Furthermore, inclusion of male individuals only did not allow the assessment of potential sex differences. Finally, due to our relatively small sample size, our study might not have had the power to detect smaller effect sizes, even though this limitation is partially counterbalanced by the cross-over design. Furthermore, the placebo-controlled study design enabled us to distinguish the effects caused by LPS from the effects caused by the injection itself. Also, the high temporal resolution over the first 6 h made it possible to dissect dynamic effects that were of relatively short duration. Finally, the analysis over time of several metabolites along the kynurenine pathway in human subjects allowed us to shed light onto the chronological relationship between activation of enzymes and changes in metabolite levels, emphasizing the importance of this study design for the analysis of pathways and metabolites in a dynamic equilibrium. Indeed, complex effects on metabolite kinetics might be lost if studying too few timepoints and/or single metabolites.

## Conclusion

In summary, our results confirm that LPS induces the kynurenine pathway in humans and that this activation lasts for at least 48 h post LPS injection. Future studies will be important to investigate whether LPS induction of the kynurenine pathway is associated with depressive symptoms and/or cognitive deficits in experimental human endotoxemia.

## Supplementary Information

**Additional file 1: Supplementary Figure 1.** Relationship between inflammatory markers and the metabolite ratios. Each dot represents one measurement (i.e. one individual, one timepoint). Light grey lines indicate the mean association for each individual. The black line shows the predicted mean calculated from the mixed effects model. **Supplementary Figure 2.** Associations between metabolite ratios and CRP or IL-6 over time. The size of the circles indicates the strength of the association (-log_10_p), the color indicates the direction of the association (red: positive, blue: negative). * <0.0083, + *p*<0.05. KYNA: Kynureninic acid, QUIN: Quinolinic Acid. **Supplementary Table 1.** Vital parameters presented as mean and standard deviation (SD). The maximum increase is defined as the largest positive difference between any timepoint and the baseline, the maximum decrease is defined as the largest negative difference between any timepoint and the baseline.

## Data Availability

The datasets used and/or analyzed during the current study are available from the corresponding author on reasonable request.

## Abbreviations

3-HK: 3-hydroxykynurenine
CRP: C-reactive protein
CV: Coefficients of variation
IDO1: Indoleamine-pyrrole 2,3-dioxygenase
IFN: Interferon
IL: Interleukin
IS: Internal standards
LPS: Lipopolysaccharide
KATs: Kynurenine aminotransferases
KMO: Kynurenine monooxygenase
KYNA: Kynurenic acid
KYNU: Kynureninase
MRM: Multiple reaction monitoring
TDO: Tryptophan 2,3-dioxygenase
TNF: Tumor necrosis factor
QC: Quality controls
QUIN: Quinolinic acid
