# Supplementary material for: Intravenous administration of LPS activates the kynurenine pathway in healthy male human subjects: a prospective placebo-controlled cross-over trial
Source: J Neuroinflammation. 2021 Jul 17;18:158. doi: 10.1186/s12974-021-02196-x (PMC8286561; doi:10.1186/s12974-021-02196-x)
Supplement: Supplementary file 1 — Additional file 1: Supplementary Figure 1. Relationship between inflammatory markers and the metabolite ratios. Each dot represents one measurement (i.e. one individual, one timepoint). Light grey lines indicate the mean association for each individual. The black line shows the predicted mean calculated from the mixed effects model. Supplementary Figure 2. Associations between metabolite ratios and CRP or IL-6 over time. The size of the circles indicates the strength of the association (-log10p), the color indicates the direction of the association (red: positive, blue: negative). * <0.0083, + p<0.05. KYNA: Kynureninic acid, QUIN: Quinolinic Acid. Supplementary Table 1. Vital parameters presented as mean and standard deviation (SD). The maximum increase is defined as the largest positive difference between any timepoint and the baseline, the maximum decrease is defined as the largest negative difference between any timepoint and the baseline. [file 12974_2021_2196_MOESM1_ESM.docx]

**Supplementary Material**

**Supplementary Figure 1.** Relationship between inflammatory markers and the metabolite ratios. Each dot represents one measurement (i.e. one individual, one timepoint). Light grey lines indicate the mean association for each individual. The black line shows the predicted mean calculated from the mixed effects model.


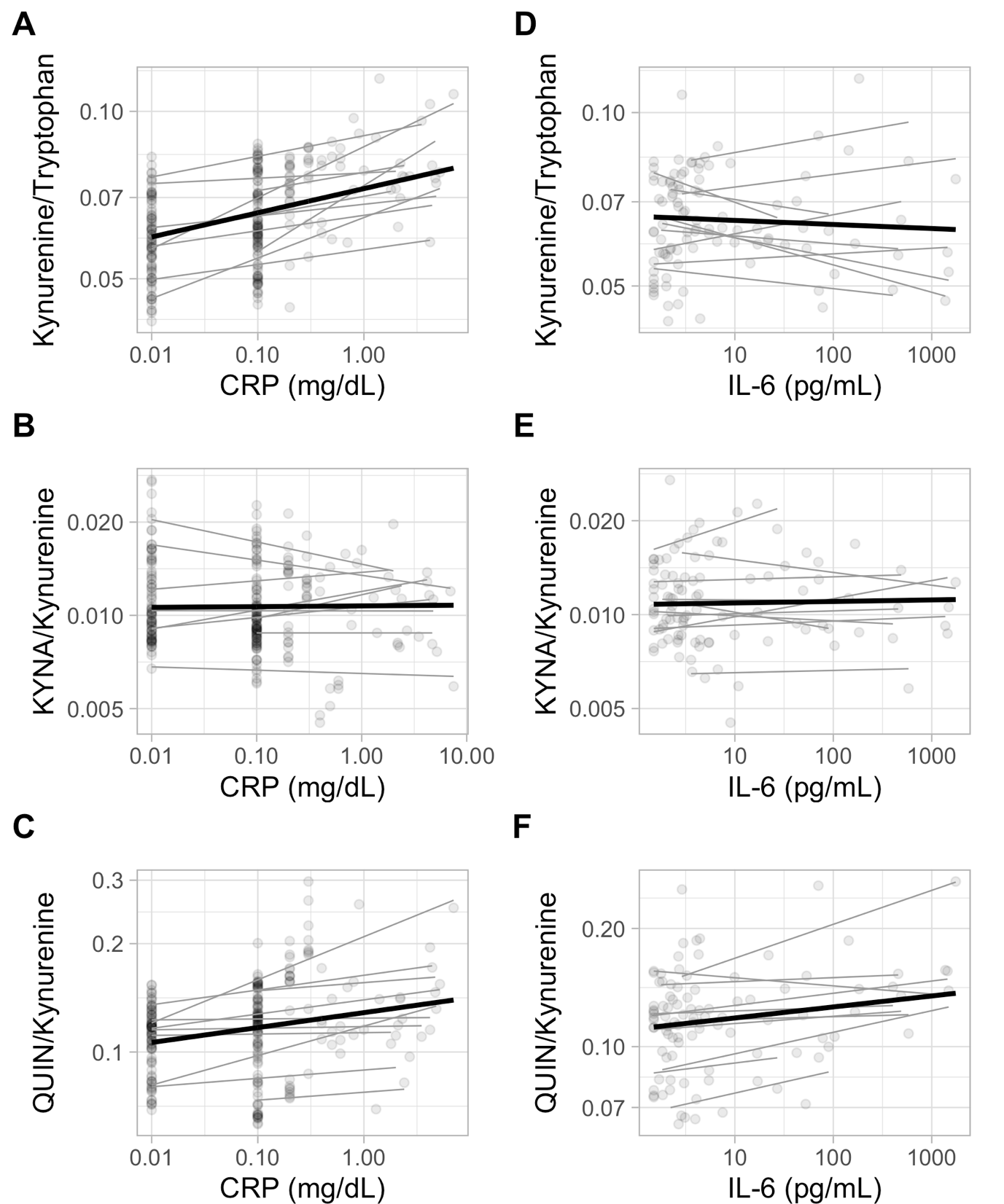


**Supplementary Figure 2.** Associations between metabolite ratios and CRP or IL-6 over time. The size of the circles indicates the strength of the association (-log_10_p), the color indicates the direction of the association (red: positive, blue: negative). * <0.0083, + p<0.05. KYNA: Kynureninic acid, QUIN: Quinolinic Acid.


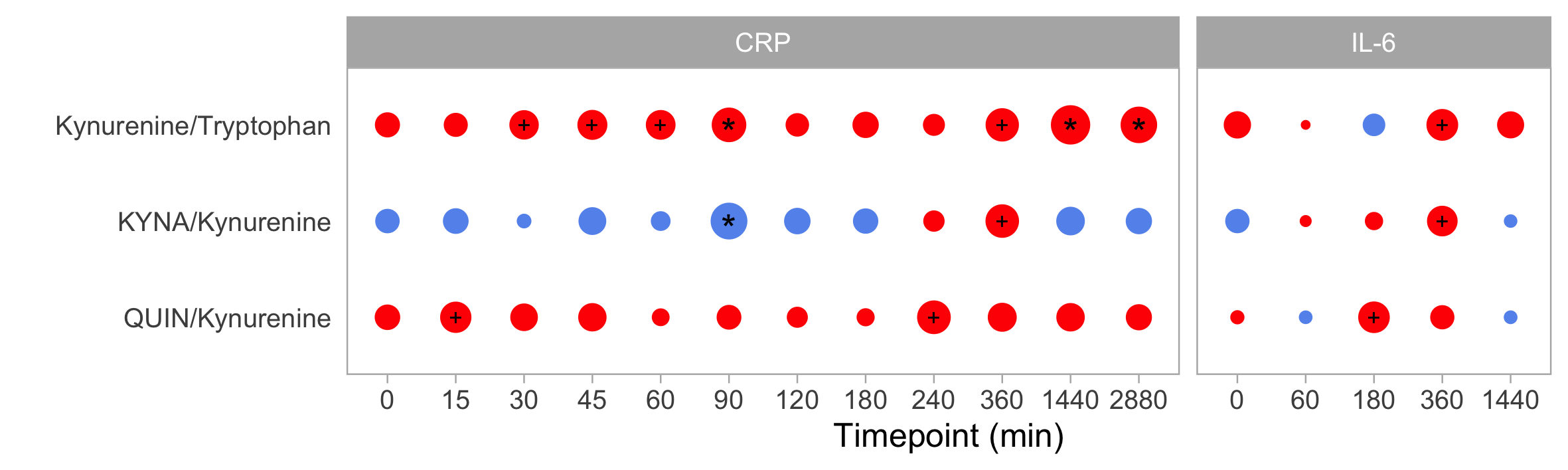


**Supplementary Table 1**.

Vital parameters presented as mean and standard deviation (SD). The maximum increase is defined as the largest positive difference between any timepoint and the baseline, the maximum decrease is defined as the largest negative difference between any timepoint and the baseline.

|  | **LPS** | **Placebo** |
| --- | --- | --- |
| **Baseline** | mean (SD) | mean (SD) |
| Systolic blood pressure (mmhg) | 132 (9) | 129 (13) |
| Diastolic blood pressure (mmhg) | 74 (11) | 77 (13) |
| Heart rate (beats per minute) | 72 (7) | 68 (10) |
| Temperature (°C) | 36 (0.34) | 36 (0.23) |
|  |  |  |
| **Maximum increase** |  |  |
| Systolic blood pressure (mmhg) | 13.7 (7.7) | 9.9 (10.3) |
| Diastolic blood pressure (mmhg) | 15 (8) | 9.8 (14.1) |
| Heart rate (beats per minute) | 32.5 (11.5) | 11.9 (8.8) |
| Temperature (°C) | 1.39 (0.8) | 0.17 (0.17) |
|  |  |  |
| **Maximum decrease** |  |  |
| Systolic blood pressure (mmhg) | -29.6 (7.1) | -19 (9.7) |
| Diastolic blood pressure (mmhg) | -31.8 (10.5) | -20.2 (11) |
| Heart rate (beats per minute) | -11.3 (8.3) | -15 (6.6) |
| Temperature (°C) | -0.07 (0.1) | -0.21 (0.29) |
